# Growth factors and osseointegration in dental implants - A review

**DOI:** 10.6026/973206300212464

**Published:** 2025-08-31

**Authors:** Neelam Chandwani, Preeti Nigotia, Sandeep Kumar Jain, Nilesh Joshi, Mridula Joshi, Rashmi Laddha

**Affiliations:** 1 Department of Dentistry, All India Institute of Medical Sciences, Nagpur, Maharashtra, India; 2 Department of Medicine, SRVS Medical College, Shivpuri, Madhya Pradesh, India; 3 Department of Medicine, Chhindwara Institute of Medical Sciences, Madhya Pradesh, India; 4 Department of Periodontology, Bharati Vidyapeeth (Deemed to be University) Dental College, Navi Mumbai, India; 5 Department of Prosthodontics, Bharati Vidyapeeth (Deemed to be University) Dental College, Navi Mumbai, India; 6 Department of Periodontology, Dr. RR Kambe Dental College and Hospital, Akola, Maharashtra, India

**Keywords:** Growth factors, osseointegration, dental implants, bone regeneration, platelet-rich fibrin (PRF), bone morphogenetic proteins (BMP)

## Abstract

Osseointegration is essential for the long-term success of dental implants. However, achieving consistent and rapid integration remains challenging. Growth factors like PDGF, BMPs, TGF-β, IGF, VEGF, PRP and PRF have shown potential to enhance bone healing and implant stability. These agents promote osteoblast activity, angiogenesis and matrix formation, accelerating peri-implant bone regeneration. Delivery methods such as platelet concentrates, surface coatings and biomaterial integration have demonstrated promising outcomes. Therefore, it is of interest to review summarizing the biological rationale, clinical evidence, application techniques and future prospects of growth factor use in implant dentistry.

## Background:

Dental implants have revolutionized prosthetic dentistry by offering a stable and long-term solution for the replacement of missing teeth [1]. The principal determinant of the success of an implant is whether or not it can achieve and sustain osseointegration a direct, functional and structural link between the surface of the implant and the bone surrounding it [2]. This biological process is necessary for the mechanical stability and lifespan of dental implants [3]. Nonetheless, obtaining predictable and fast osseointegration remains a clinical problem, especially in compromised bone quality, medically compromised cases, or when immediate loading protocols are involved [4]. Owing to the complexity of the process, osseointegration involves several phases of biological nature, such as inflammation, recruitment, proliferation, angiogenesis, matrix deposition and remodeling of bone [5]. These phases are strictly regulated by several molecular cues, among which growth factors represent a key component [6]. Growth factors are endogenous proteins that play a role in cellular processes including chemotaxis, proliferation, differentiation and extracellular matrix production [7]. They have been firmly established in wound healing and bone repair in the fields of medicine and dentistry [8]. Over the last few decades, there has been increasing interest in using exogenous growth factors or autologous platelet-rich plasma to control and maximize the biological condition around implants [9]. Adjunctive treatments using platelet-derived growth factor (PDGF), bone morphogenetic proteins (BMPs), transforming growth factor-beta (TGF-β), insulin-like growth factor (IGF), vascular endothelial growth factor (VEGF), platelet-rich plasma (PRP) and platelet-rich fibrin (PRF) have also been promising to enhance bone formation, increase implant stability and decrease overall healing time [10]. Therefore, it is of interest to describe the role of growth factors in promoting osseointegration of dental implants by examining their biological mechanisms, summarizing existing experimental and clinical evidence, discussing application methods and highlighting current challenges, limitations and future directions in research and clinical practice.

## Biological basis of osseointegration:

Osseointegration is the direct structural and functional bond between bone alive and the surface of a load-carrying implant and is an on-going and intricate biological process involving several cellular and molecular events that provide the firm anchorage of the implant to jawbone. It is essential that the osseointegration biological processes are comprehended in an attempt to optimize implant success and investigate adjunctive treatment, like growth factors, to facilitate this process. Osseointegration can be broadly divided into three intersecting phases: the inflammatory, the proliferative and remodeling phases. The implant act is followed by surgical trauma, which invokes inflammatory response involving immune cell recruitment and release of growth factors and cytokines. It provides a platform for tissue repair by eliminating debris and requesting the delivery of regenerative cells. In the proliferative stage, osteoblasts are formed through the process of differentiation of mesenchymal stem cells, which start producing fresh bone matrix on the surface of the implant. Angiogenesis, or new vessel formation, is essential in this stage to provide oxygen and nutrition to the growing tissue. The quantity and quality of this new bone will determine the major stability of the implant [11]. Remodeling stage includes maturation and accommodation of bone tissue to mechanical loads. Immature bone is resorbed by osteoclasts and mature lamellar bone is deposited by osteoblasts to form a strong and highly functional bone-implant interface able to sustain functional loads. Phases are governed by a multitude of signaling molecules, growth factors acting as master modulators in stimulating cell migration, proliferation, differentiation and extracellular matrix deposition. The communication between the implant surface characteristics and the biological environment also controls the extent and speed of osseointegration. Biomaterial developments have been towards the modification of implant surfaces for enhancing cellular response and for more accelerated osseointegration. Biological adjuncts like growth factors provide a potential means for augmenting the inherent regenerative potential of bone and soft tissue, to potentially shorten healing time and enhance clinical result [12].

## Growth factors in bone regeneration:

Growth factors are naturally produced proteins that govern cellular processes concerned with tissue healing and regeneration. During bone regeneration and healing, growth factors behave as signaling factors to regulate osteogenic cell proliferation, differentiation and activity, angiogenesis and extracellular matrix production. These growth factors are secreted from different cell types, such as platelets, macrophages, osteoblasts and endothelial cells and act by binding to their specific cell surface receptors to induce intracellular cascade signaling. Coordination among different growth factors facilitates the temporal development of the cascade of bone healing. Growth factors have been the focal point of much attention in dental implantology because of their ability to accelerate and enhance osseointegration [13]. By improved cellular recruitment and activity in the area of implantation, they promote faster bone regeneration and improved implant integration. Some of the major growth factors involved in bone regrowth are platelet-derived growth factor (PDGF), bone morphogenetic proteins (BMPs), transforming growth factor-beta (TGF-β), insulin-like growth factor (IGF) and vascular endothelial growth factor (VEGF). These molecules initiate processes including chemotaxis of the progenitor cells, osteoblast proliferation, differentiation of the mesenchymal stem cells into bone cells and angiogenesis. Moreover, the introduction of platelet concentrates like platelet-rich plasma (PRP) and platelet-rich fibrin (PRF) has made it possible to use autologous growth factors at the clinical level, offering an economical and biocompatible means for augmentation of bone and healing of soft tissue. The incorporation of growth factors into implant protocols tries to overcome the biological constraints of healing, particularly in challenging situations and enhance implant success and patient outcomes [14].

## Types of growth factors used in implant dentistry:

Different growth factors were investigated and applied in implant dentistry because of their specific roles in bone and soft tissue augmentation. Platelet-Derived Growth Factor (PDGF) is a strong mitogen that attracts osteoblasts and mesenchymal stem cells to the site of the implant and promotes their proliferation. It participates in angiogenesis and matrix remodeling. Bone Morphogenetic Proteins (BMPs) BMP-2 and BMP-7 belong to the transforming growth factor-beta superfamily and play a significant role in inducing osteogenic differentiation of mesenchymal stem cells. BMPs have been extensively researched for their potential to cause de novo bone formation and are incorporated into various bone graft substitutes to enhance implant integration. Transforming Growth Factor-Beta (TGF-β) controls cell proliferation and differentiation and affects the production of components of the extracellular matrix. It is also involved in progenitor cell recruitment during bone repair. Insulin-like Growth Factor (IGF) stimulates osteoblast proliferation and differentiation and increases collagen synthesis, crucial for the bone matrix [15]. Vascular Endothelial Growth Factor (VEGF) is among many associated with angiogenesis and induces the development of new vessels, critical for oxygen and nutrient delivery throughout the bone healing process. Platelet-Rich Plasma (PRP) is defined as an autologous platelet-rich plasma concentration that contains a high level of growth factors such as PDGF, TGF-β and VEGF. Platelet-Rich Fibrin (PRF) is a second-generation platelet concentrate that elaborates further the concept of developing a fibrin matrix and releasing growth factors in a slow, long-lasting manner to regenerate bone and soft tissues. Concentrated Growth Factor (CGF) is a more recent platelet concentrate with a high-density fibrin matrix and greater concentrations of growth factors than PRP and PRF and has the potential to increase bone regeneration and osseointegration of implants [16].

## Evidence supporting the use of growth factors in osseointegration:

Extensive research has explored the ability of growth factors to enhance osseointegration on the basis of *in vitro*, animal and human models. Time after time, *in vitro* experiments have demonstrated that growth factors such as PDGF, BMPs and TGF-β significantly enhance the proliferation, differentiation and mineralization of osteoblasts. Particularly, BMP-2 stimulates osteogenic gene expression and matrix deposition in mesenchymal stem cells, whereas platelet concentrates such as PRP and PRF stimulate cell migration and early healing responses. There is also additional evidence from animal studies, where preclinical models using rabbits and dogs demonstrate greater bone-to-implant contact (BIC) and new bone formation when implants are conditioned with or coated using growth factor-enriched materials. BMP-2, however, has been particularly successful in speeding up bone regeneration and enhancing implant stability in such models. Growth factor application has also been described with successful outcomes in clinical trials, including decreased healing times, greater values for the implant stability quotient (ISQ), better marginal bone preservation and better survival rates [17]. The use of PRF has, for example, been attributed to faster healing of soft tissues and better bone regeneration after surgery. However, heterogeneity among study designs, patient populations, growth factor preparation protocols and outcome measures makes it difficult to draw uniform conclusions. Comparative analyses, such as systematic reviews and meta-analyses, support the efficacy of growth factors, particularly in immediate implant placement or compromised bone conditions. Nevertheless, challenges remain because of varying clinical protocols and lack of standardized delivery methods. Overall, although current literature has strong biological rationale and promising clinical evidence to support the use of growth factors in implant dentistry, additional high-quality, well-standardized clinical trials are crucial for establishing their best application strategies and confirming long-term efficacy [18].

## Application methods of growth factors in implant dentistry:

Clinical effectiveness of growth factors in enhancing osseointegration is not only a function of the growth factor employed but also of the manner in which it is delivered to the site of the implant. Various strategies have now been developed to optimize their therapeutic efficacy and bioavailability. The simplest of these is direct application, where the growth factors are implanted on the surface of the implant or on the surrounding bone during surgery. This is done by submerging implants in growth factor solutions or placing enhanced gel or membranes in the site. Sited, though, the approach has transient duration as a result of rapid breakdown. More biologically retained is the application of autologous platelet concentrates that encompass PRP, PRF and CGF. These consist of a dense blend of endogenous growth factors encased within a fibrin matrix that permits controlled release as well as facilitating cell proliferation and angiogenesis [19]. These are administered as gels, membranes, or blended with bone grafts for an improved regenerative response. One of the recent strategies is loading growth factors into biomaterials like bone graft substitutes, scaffolds, or hydrogels, which makes delivery controlled and sustained. BMPs loaded in collagen or polymers are prolonged in their bioactive state for extended periods of time and produce localized therapeutic effects. Besides, immobilization of growth factors onto implant surfaces towards the development of bioactive implants that induce osteogenesis at implantation has been investigated. It involves immobilization of proteins such as BMPs onto titanium surfaces but with close control of release kinetics. Combined therapies with growth factors and stem cells, bone graft, or scaffolds all have a synergistic effect providing structural support, cellular material and biochemical cues at the same time. These methods of application, when carefully chosen and utilized, have a dramatic impact on clinical success-promoting faster healing, enhanced bone quality, enhanced implant stability and greater success rates. Patient variation and protocol variation emphasize, however, the need for cautious clinical judgment and standardization [20].

## Impact on clinical outcomes:

Addition of growth factors in dental implantology has positively influenced clinical results by enhancing hard and soft tissue healing. Among the most important advantages observed is shortening of the healing time and hence a possibility for earlier loading of the implant as well as higher patient satisfaction. BMPs and PDGF are growth factors that stimulate osteoblast activity and hasten bone remodeling, thus contributing to quicker osseointegration. Another important implication is the rise in implant stability, as measured by Implant Stability Quotient (ISQ) values. Research on platelet concentrate use such as PRF and CGF has reported very high ISQ values during the initial healing stages, reflecting increased primary and secondary stability. Additionally, marginal bone level maintenance around implants has been observed to be enhanced with growth factor use [21]. Through modulation of inflammation and angiogenesis stimulation, growth factors maintain bone volume and minimize crestal bone loss over time. Soft tissue healing and esthetics are also improved by growth factor-accumulated protocols. PRF and PRP facilitate more rapid epithelialization and more extensive soft tissue sculpting, both critical to anterior implant esthetic success. Growth factor delivery also minimizes peri-implant issues such as dehiscence or delayed healing, especially in medically compromised patients or at bone-compromised locations. Clinical benefits are observed most significantly in complicated cases, such as immediate placement, sinus augmentation and ridge preservation therapy. In these scenarios, growth factors deliver a biological signal that increases successful implant integration when traditional methods may fail or take longer to heal. Overall, the application of growth factors in dental implant therapy suggests more consistent outcomes, shorter treatment periods and enhanced patient-satisfaction rates [22]. However, to be completely effective by applying these effects in usual practice, practitioners must remember certain patient considerations, cost implications and the lack of standard application protocols available to date.

## Discussion:

Growth factor application in dental implantology is a potential development for speeding up osseointegration and enhancing success rates of implants. The literature proves that growth factors including PDGF, BMPs, TGF-β, IGF and VEGF are critical factors in bone healing and soft tissue repair through the modulation of cell proliferation, differentiation, angiogenesis and matrix production. Autologous platelet concentrates such as PRP, PRF and CGF are in the form of readily available delivery systems which augment the biological activity by sustained release of such bioactive molecules [23]. *in vitro* and animal study evidence on a consistent basis supports the rationale for the use of growth factors by biological means, with clinical findings of increased stability of implants, healing time reduction and improvement in bone quality. Yet, in spite of these promising results, the implementation of growth factor therapies into day-to-day clinical practice is not without challenges. Inconsistency in preparation protocols, dosages and application methods makes standardization of treatment difficult and produces heterogenous outcomes [24]. Additionally, the cost and availability of some recombinant growth factors might restrict their widespread application. The absence of long-term clinical trials and standardized guidelines further limits firm conclusions about optimal protocols and safety profiles. Combination regimens with growth factors, bone grafting and stem cells are synergistic but require further study in order to define standardized protocols [25]. In general, although the therapeutic potential of growth factors is promising for enhancing osseointegration, clinically their application must be employed cautiously. On-going research involving well-designed randomized controlled trials and follow-up over the long term will be required to validate their effectiveness, standardize application procedures and estimate cost-effectiveness. A multidisciplinary approach integrating biomaterials science, molecular biology and clinical dentistry is likely to unlock more therapeutic options in dental implantology.

## Limitations and considerations:

While the promise of growth factors in osseointegration is bright, their clinical use is marred by several challenges. They are expensive for some preparations of growth factors, heterogeneous in preparation and use and potentially subject to an immunogenic reaction, particularly when recombinant proteins are used. Lack of standardized clinical protocols and guidelines also keeps it from becoming a universally used and equally outcome-producing procedure. To address these issues, further well-designed randomized controlled trials with longer follow-up are required to completely evaluate the efficacy, safety and cost-effectiveness of growth factors in routine dental implant therapy.

## Conclusion:

Growth factors play a significant role in enhancing osseointegration by promoting cellular activity and tissue regeneration around dental implants. While data supports their benefits in improving implant stability and healing, the lack of standardized protocols and high variability in application limit widespread clinical use. Hence, future research should focus on well-structured clinical trials to establish optimal guidelines and assess long-term efficacy and safety.
